# Targeting cardiac hypertrophy through a nuclear co‐repressor

**DOI:** 10.15252/emmm.201911297

**Published:** 2019-10-17

**Authors:** Andrea Grund, Joerg Heineke

**Affiliations:** ^1^ Department of Cardiovascular Research European Center for Angioscience (ECAS) Medical Faculty Mannheim German Centre for Cardiovascular Research (DZHK, partner site Heidelberg/Mannheim) University of Heidelberg Mannheim Germany; ^2^ Department of Cardiovascular Research Medical Faculty Mannheim University of Heidelberg Mannheim Germany

**Keywords:** Cardiovascular System

## Abstract

Heart failure entails the inability of the heart to pump blood to vital organs. One of the main risk factors for heart failure is the development of pathological hypertrophy. In this issue of *EMBO Molecular Medicine*, Li and coworkers show that NCoR1, a co‐repressor of transcription factors, inhibits the transcriptional activity of MEF2 by stabilizing its complex with class II HDACs. By this mechanism, NCoR1 was identified as potent inhibitor of pathological cardiac hypertrophy and dysfunction.

Chronic heart failure is becoming increasingly prevalent and is still associated with high mortality rates comparable to that of many cancers (Mamas *et al*, 2017). It develops as sequel of myocardial infarction, but also due to chronic cardiac pressure overload, which results, for example, from narrowing of the aortic valve or from long‐standing arterial hypertension (Heineke & Molkentin, 2006). To maintain cardiac output and blood pressure, neurohormones are released and cardiac growth is enhanced. Because cardiomyocytes lose their ability to proliferate shortly after birth, cardiomyocyte growth is the result of cellular enlargement, i.e., cardiomyocyte hypertrophy. Although initially compensatory, cardiac hypertrophy in response to pathological stimulation is frequently associated with left ventricular dysfunction and with increased mortality. Therefore, it is a valuable therapeutic target and anti‐hypertrophic therapies are expected to improve the outcome of heart failure. Current therapies such as beta‐blockers are designed to interfere with the neurohormonal system, but can only partially reverse hypertrophy or improve heart function. Additional strategies that target central signaling mechanisms within cardiac myocytes are needed. Prohypertrophic signaling circuits trigger a profound change in gene expression, which includes activation of a so‐called *embryonic gene program* with re‐expression of the fetal cardiac genes *Acta1*,* Nppa,* and *Nppb,* as well as prohypertrophic genes. Among the transcription factors that play a crucial role in mediating heart development and cardiac reprogramming are GATA transcription factors, NKX2.5 and myocyte enhancer factor 2 (MEF2; Heineke & Molkentin, 2006). The MEF2 family of transcription factors are key regulators of cardiac development and induce cardiac hypertrophy during postnatal cardiac overload. MEF2 is suppressed by epigenetic regulators called class II histone deacetylases (HDAC, including HDAC4, HDAC5, and HDAC7), which act through de‐acetylating lysine residues of histones and inducing a condensed and inaccessible chromatin state. Cardiomyocyte class II HDACs respond to prohypertrophic stimuli as they get phosphorylated by CamKII, leading to their export from the nucleus and the expression of MEF2‐dependent prohypertrophic genes (Backs & Olson, 2006). The recruitment of chromatin‐modifying enzymes by transcription factors is often mediated by co‐regulators, which can be co‐activators or co‐repressors. Co‐activators recruit histone acetyltransferases to activate transcription, while co‐repressors suppress gene transcription by linking them to HDACs. In this issue of *EMBO Molecular Medicine,* Li *et al* report a previously unrecognized role of the co‐repressor nuclear receptor co‐repressor 1 (NCoR1) in the heart as inhibitor of cardiac hypertrophy (Li *et al*, 2019). NCoR1 is a large protein of around 270 kDa that is composed of multiple domains and acts in general as a scaffold protein interacting with multiple partners. It is highly homologous to the silencing mediator of retinoic acid and thyroid hormone receptor (SMRT, also known as NCoR2) (Mottis *et al*, 2013). NCoR1 exhibits specialized repressive domains (RD) and SANT domains in its N‐terminal end, which enable interaction with HDAC3 (a class I HDAC) and HDACs 4, 5, and 7, and mediates the repressive activity of the protein. At the C‐terminal end are three RID domains that were shown to bind nuclear receptors and various transcription factors. NCoR1 has been shown to inhibit growth and mitochondrial biogenesis in skeletal muscle (Yamamoto *et al*, 2011). In their study, Li *et al* present new insight into the functions of NCoR1 in cardiomyocytes during pathological overload and hypertrophic cardiomyopathy. Interestingly, under these conditions, NCoR1 becomes upregulated in the myocardium. The authors generated mutant mice with a cardiomyocyte‐specific knock‐out of NCoR1, which develop myocardial hypertrophy and impaired cardiac function at the age of 10 months, as well as in pathological cardiac overload due to abdominal aortic constriction (ACC). Gene‐expression profiling of the NCoR1‐deficient mice hearts uncovered the upregulation of MEF2‐dependent genes. Similarly, knocking down NCoR1 in isolated primary cardiomyocytes triggered an increased hypertrophic response after neurohormonal stimulation. Interestingly, this response was completely abrogated by concomitant knock‐down of MEF2A or MEF2D (the main MEF2 genes expressed in the adult heart), indicating that increased hypertrophy after inactivation of NCoR1 was mediated by MEF2. Indeed, the authors further demonstrated that NCoR1 acts in a complex together with MEF2 and HDAC4 and 5. In this complex, NCoR1 suppresses MEF2‐dependent prohypertrophic gene expression by stabilizing the association between MEF2 and HDACs, and even preventing their nuclear export after prohypertrophic stimulation. Within NCoR1, the interacting domain was mapped to the region of the RIDs, and overexpression of the RIDs region by an adeno‐associated virus 9‐based gene‐therapeutic approach was sufficient to inhibit hypertrophy and cardiac dysfunction in mice.

As many good studies do, this work raises new questions. In skeletal muscle, NCoR1 affects energy metabolism, while in macrophages it suppresses inflammation (Mottis *et al*, 2013). Is that also the case in the heart? This will need to be addressed in future studies. Most importantly, it remains unclear how NCoR1 itself is regulated. Are there circumstances that lead to its downregulation? In muscle for example, chronic high‐fat diet or aging triggers NCoR1 downregulation. Could this decrease be responsible for cardiac hypertrophy, which is associated with both conditions? NCoR1 upregulation during pathological overload and hypertrophic cardiomyopathy is likely a compensatory mechanism to prevent cardiac over‐growth. On the other hand, how is growth of the heart possible with even higher NCoR1 levels, which interfere with HDAC export? Similarly to class II HDACs, NCoRI can be phosphorylated, which leads to its nuclear export. Could this enable hypertrophy during overload? If yes, which kinase could be responsible? Beside nuclear export, phosphorylation of the homologous NCoR2 by the prohypertrophic MAP kinase ERK2 can lead to destabilization of the co‐repressor complex (Mottis *et al*, 2013). Clearly, a better understanding of the co‐repressor complex and its modification by hypertrophic signaling could lead to novel therapeutic strategies, perhaps involving pharmacological interventions by small molecules to inhibit protein–protein interactions. This could be more feasible than cardiac gene therapy, which has not yet been successfully established at a larger scale in humans.

Taken together, the present work from Li *et al* (summarized in Fig 1) deepens our knowledge on cardiac growth regulation, and the NCoR1/MEF2/class II HDACs axis may in the future be a target for new therapies to inhibit pathological cardiac hypertrophy.

**Figure 1:**
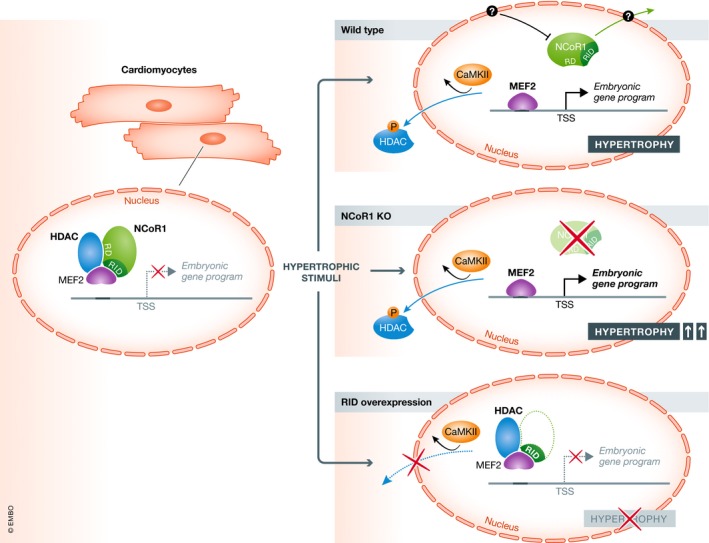
In cardiomyocytes, NCoR1 and class II HDACs bind the transcription factor MEF2 and block its activity, cardiomyocyte hypertrophy, and the expression of embryonic genes (left panel) Prohypertrophic stimulation of WT (wild‐type) cardiomyocytes leads to phosphorylation of HDACs by CaMKII and their translocation to the cytosol, which promotes the activation of MEF2. The mechanism of how NCoR1 releases MEF2, for example, through posttranslational modification and translocation of NCoR1, remains unclear (upper right panel). Likewise, prohypertrophic stimulation of cardiomyocytes lacking NCoR1 leads to phosphorylation and translocation of HDACs and to activation of MEF2 (middle right panel). Overexpression of the receptor interaction domains (RID) of NCoR1 in cardiomyocytes is sufficient to inhibit MEF2 activity and cardiomyocyte hypertrophy (lower right panel). TSS—transcription start site.
